# *In situ* detection of the eIF4F translation initiation complex in mammalian cells and tissues

**DOI:** 10.1016/j.xpro.2021.100621

**Published:** 2021-06-23

**Authors:** Shensi Shen, Isabelle Girault, Hélène Malka-Mahieu, Caroline Robert, Stéphan Vagner

**Affiliations:** 1 Institute of Thoracic Oncology, Department of Thoracic Surgery, West China Hospital, Sichuan University, Chengdu, China; 2 Singapore-Sichuan Frontier Medical Center, Sichuan University, Chengdu, China; 3 INSERM U981, Gustave Roussy Cancer Campus, Villejuif, France; 4 Institut Curie, Université PSL, CNRS UMR3348, INSERM U1278, 91400 Orsay, France; 5 Université Paris-Saclay, CNRS UMR3348, INSERM U1278, 91400 Orsay, France; 6 Equipe labellisée Ligue contre le cancer, Kremlin-Bicetre, France; 7 Université Paris-Saclay, Kremlin-Bicetre, France; 8 Gustave Roussy Cancer Campus, Dermato-Oncology, Villejuif, France

**Keywords:** Cell culture, Cell-based Assays, Microscopy, Molecular Biology

## Abstract

The eukaryotic translation initiation complex eIF4F plays an important role in gene expression. The methods that are used to monitor the formation of the eIF4F complex are usually indirect and provide no information on its subcellular localization. This protocol describes a proximity ligation assay-based procedure allowing the direct *in situ* visualization of the eIF4F complex, as well as its absolute quantification per cell using adapted image analysis software.

For complete details on the use and execution of this protocol, please refer to Boussemart et al. (2014).

## Highlights

• *In situ* detection of the interactions between eIF4E and either eIF4G or 4EBP1
• Co-localization of eIF4F complex and cellular organelles
• Complete 3D quantification of the interactions within the eIF4F complex
• Evaluation of eIF4F complex formation upon clinically relevant treatment perturbation

## Graphical abstract

**Figure:**
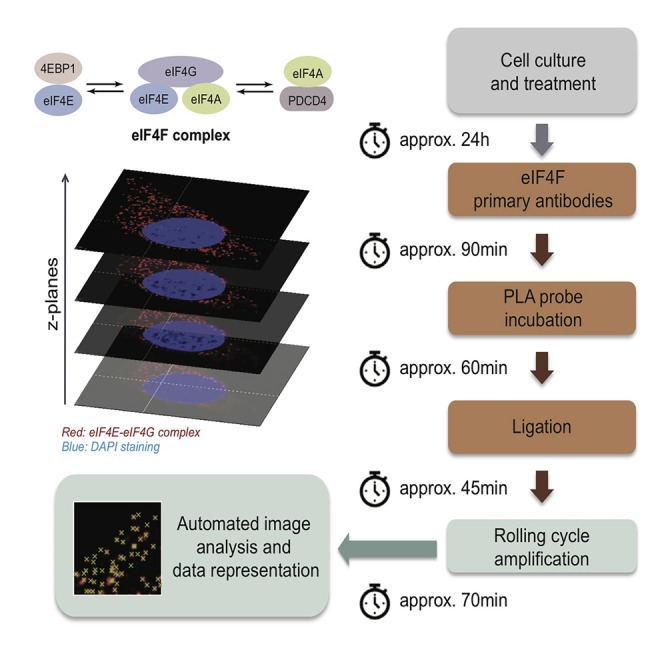

mRNA translation initiation is regulated by eIF4F, a protein complex consisting of the eIF4E cap binding protein, the eIF4G scaffolding protein, and the eIF4A DEAD-box RNA helicase. The formation of this complex is inhibited by 4EBP1 which directly interacts with eIF4E. We have recently described a procedure based on proximity ligation assay (PLA) to detect the eIF4E-eIF4G and eIF4E-4EBP1 interactions at the single-molecule level in cell lines as well as formalin-fixed embedded (FFPE) mouse and human tumors.

## Before you begin

This protocol below describes the in situ detection of the eIF4F complex in the A375 human melanoma cell line. However, we have also used this protocol in multiple other cell lines, i.e., human melanoma, ovarian and breast cancer cell lines and murine melanoma-derived cell lines (Boussemart et al., 2014; Cerezo et al., 2018).

### Reagent setup

**Timing: approximately 2h**

**Choice of primary antibodies:** The two primary antibodies should be IgG-class, with high affinity purity for the protein. Both monoclonal and polyclonal are applicable. For the detection of protein-protein interactions, the two primary antibodies must be raised in two different species and must bind to the proteins (with a maximum distance of 40 nm) under the same fixation-permeabilization conditions.

1. **Determination of the limit dilution of the primary antibodies:** The concentration of the primary antibodies has to be titrated in each used cell lines. As shown in Figure 2E, one can start with a saturated concentration (1:50 dilution in most cases) and then perform a serial dilution of the antibodies until the number of quantified PLA complex start to decrease. In our settings, we found that eIF4E and eIF4G antibodies can be used at 1:500 dilution.
2. **Determine the method of fixation-permeabilization:** The fixation and permeabilization conditions have to be optimized. Use the recommendation from the vendor, or test the immunohistochemistry normally used conditions (Methanol/Acetone (V/V), PFA 4%/PBS-Tween20 0.2%, PFA 4%/PBS-Triton X100 0.1%). We have improved the fixation method in this protocol by using 4% PFA.
3. **Paraformaldehyde 4% (vol/vol):** Make a 4 times dilution of PFA (16%) in 1**×** PBS. Store the solution at −20°C for up to two months. The 4% PFA must be recovered during 30 min to room temperature before use.

**Table:** 

4% PFA	10 mL 16% PFA stock solution, fill up to 40 mL with 1**×** PBS solution.

4. **PBS 1×, Tween-20 0.2%:** Add 40 μL of Tween-20 in 20 mL of 1**×** PBS. Store the solution at 4°C for up to one month.

**Table:** 

0.2% Tween-20	40 μL of 100% Tween-20 stock solution, fill up to 20 mL with 1**×** PBS solution.

5. **Probe solution**: Dilute each PLA probe (1:5) in antibody diluent.
6. **Ligation reaction buffer**: Dilute the ligase (40**×**) in the ligation solution (5**×**) with milliQ water immediately before use.
7. **Polymerase**: Dilute the polymerase (80**×**) in the amplification solution (5**×**) with milliQ water immediately before use.
8. **Wash buffer A**: Dissolve one pouch in distilled water to a final volume of 1 L and store at 4°C. Alternatively, buffer A can be prepared by dissolving 8.8 g NaCl, 1.2 g Tris base in 800 mL of high purity water. Then add 0.5 mL Tween-20 and adjust pH to 7.4 using HCl. Add high purity water to a final volume of 1 L. Filter the solution through a 0.22 μm filter if the buffer is prepared in-house and store at 4°C.

**Table:** 

Reagent	Final concentration	Amount
NaCl	0.88%(w/v)	8.8g
Tris base	0.12%(w/v)	1.2g
Tween-20	0.05%(v/v)	0.5mL

9. **Wash buffer B:** Dissolve one pouch in distilled water to a final volume of 1 L and store at 4°C. Alternatively, buffer B can be prepared by dissolving 5.84 g NaCl, 4.24 g Tris base and 26.0 g Tris-HCl in 500 mL of high purity water. Adjust pH to 7.5 using HCl. Add high purity water to final volume of 1 L. Filter the solution through a 0.22 μm filter if the buffer is prepared in-house and store at 4°C.

**Table:** 

Reagent	Final concentration	Amount
NaCl	0.584%(w/v)	5.84g
Tris base	0.424%(w/v)	4.24g
Tris-HCl	2.6%(w/v)	26g

10. **Wash buffer B 1%**: Dilute 1:100 buffer B 1**×** in distilled water.
11. **Reaction volume:** Use 20 μL of total reaction volume for one cover slide (12 mm diameter).

### Equipment setup

**Timing: approximately 30 min**

12. **Microscope and imaging**: We use an Olympus scanner solution VS120 with mirror cube QUAD optimizes for imaging 4 separate channels, including DAPI, FITC, Cy3 and Cy5. Microscopy and imaging setup is controlled by OlyVIA software. For each sample we use magnification 20**×**, channel 1 (FL DAPI) wavelength 455 nm with exposure time of 3 ms, channel 2 (FL CY5) wavelength 670 nm with exposure time of 300 ms, mirror cube QUAD and 3 Z dimension with 1 μm spacing −1+1. In the case of cell line-based assay, at least three region of interest (ROI) on each slide should be acquired for statistical analysis. For 3D quantification, a minimum of 3 μm focal plane should be acquired. The image analysis method described in this protocol can also handle higher levels of image stack depending on the computation capacity. Alternative microscopic systems are also applicable. An upright fluorescent microscopy is preferred and a minimum of a 20**×** magnification objective is required for the quantification of the eIF4F complex described in this protocol. If one is not interested in the co-localization of the eIF4F complex with other intracellular organelles, at least two fluorescent channels are needed; a 455 nm UV channel for the acquisition of DAPI or similar staining probes for nuclear, and a 488 nm green filter or a 642/662 nm far red filter is required for the acquisition of PLA complexes. Automated serial scanning at vertical z-dimension is also required for an alternative microscopic system which will allow the 3D quantification of the eIF4F complex.
13. **Image analysis:** The image acquired by Olympus scanner VS120 is processed by ImageJ (Version 1.0) plugin BIOP-tool (Version 1.02). 3-dimension absolute number of translation initiation complex is analyzed with MATLAB-based FISH-Quant software (MATLAB version 7.12.0, R2011a and FISH-Quant version 1.0). For the details of the setup of the software and URL pages for download, please visit the website (https://sites.google.com/site/translationcomplexeif4f/).

## Key resources table

**Table:** 

REAGENT or RESOURCE	SOURCE	IDENTIFIER
**Antibodies**		

eIF4E antibody (clone A-10, mouse) non-replaceable reagent	Santa Cruz Biotechnology	sc-271480
eIF4A antibody (clone H-5, mouse) non-replaceable reagent	Santa Cruz Biotechnology	sc-377315
eIF4G antibody (polyclonal, rabbit) non-replaceable reagent	Cell Signaling Technology	#2498
4EBP1 antibody (polyclonal, rabbit) non-replaceable reagent	Cell Signaling Technology	#9644
GalT antibody (clone G-1, mouse)	Santa Cruz Biotechnology	sc-365577
Calnexin antibody (clone H-70, mouse)	Santa Cruz Biotechnology	sc-11397

**Critical commercial assays**		

PLA Probe Anti-Mouse PLUS	Sigma-Aldrich	DUO92001
PLA Probe Anti-Rabbit MINUS	Sigma-Aldrich	DUO92005
Blocking solution	Sigma-Aldrich	DUO92001
Antibody diluent	Sigma-Aldrich	DUO92001
PLA detection reagent	Sigma-Aldrich	DUO92013
Wash buffer A and B	Sigma-Aldrich	DUO82049-4L
Mounting oil with DAPI	Sigma-Aldrich	DUO82040-5ML
Paraformaldehyde	Electron Microscopy Sciences	#15710
Tween-20	Gibco	#14190-086
Methanol	Carlo Erba	#414814
Acetone	Carlo Erba	#508200

**Software and Algorithms**		

FISH-quant	Mueller et al. 2013	https://code.google.com/p/fish-quant/
BIOP-tool	Ecole Polytechnique Federale de Lausanne	https://http://biop.epfl.ch/TOOL_VSI_Reader.html
Demo	This paper	https://sites.google.com/site/translationcomplexeif4f
FlowJo	BD	https://www.flowjo.com/

**Other**		

Olympus scanner solution VS120	Olympus	VS120
Confocal microscope	Leica	TCS SPE
Six-well plates	TPP	92006
Twenty-four-well plates	TPP	92024
Parafilm	Sigma-Aldrich	P7793-1EA
Microscope glass slides	CML	LCSF02
Microscope cover glasses	CML	LCO12
Immedge hydrophobic barrier pen	CliniSciences	H4000
Hybridization oven	Binder	9010-0235
Tabletop centrifuge	Eppendorf	5427 R
Vortex Genie	LMS Co.	VTX-3000L
Syringe	Terumo	U-100
Shaker	Stuart	SSM1

## Materials and equipment

All the following materials are replaceable by alternative materials or equipment if suitable.

Six-well plates (TPP, cat. no. 92006)

Twenty four-well plates (TPP, cat. no. 92024)

Parafilm (Sigma-Aldrich, cat. no. P7793-1EA)

Microscope glass slides (CML, LCSF02)

Microscope cover glasses (CML, LCO12)

Immedge hydrophobic barrier pen (Clinisciences, cat. no. H4000)

Hybridization oven (Binder, cat. no. 9010-0235)

Tabletop centrifuge (Eppendorf, cat. no. 5427 R)

Vortex Genie (LMS Co., cat. no. VTX-3000L)

Syringe (TERUMO, cat. no. U-100)

Vacuum pump

Shaker (Stuart, cat. no. SSM1)

Fluorescence microscope equipment with excitation/emission filters compatible with FarRed fluorophore and nuclear stain (Olympus VS120)

Camera XM10 for image acquisition

Microscope imaging software (OlyVIA)

Computer minimum 4GB RAM

FlowJo software

The following software are non-replaceable for appropriate image analysis described in this protocol.

FISH-Quant (Mueller et al., 2013) was developed in MATLAB software (Mathwork, version 6.0 or later), the codes are available on the website (https://code.google.com/p/fish-quant/)

BIOP-tool was developed by Bioimaging and optics Platform of Ecole Polytechnique Federale de Lausanne. The code can be downloaded on the website (https://http://biop.epfl.ch/TOOL_VSI_Reader.html)

For users’ convenience, readers can find all the available software links and demo images on our website (https://sites.google.com/site/translationcomplexeif4f)

## Step-by-step method details

### Preparation of cells

**Timing: variable 24–48 h**

1. 12 mm cover glass should be placed in a 100 mm glass petri dish, autoclaved followed by drying at 37°C in a heating oven for 16h–24 h.
2. Place two sterile 12 mm cover glass in each well of a six-well culture plate, the final percentage of cell confluency should not be over 70 %.
3. Plate 2 **×** 10^5^ cells per well in 2 mL of culture medium. 

**CRITICAL:** High confluency of cells will lead to inhibition of eIF4F complex formation. Cell density is therefore an important parameter that impacts on the number of translation initiation complex in live cells. The optimal number of cells to be plated should be tested. When using different cell line models, we firstly perform a serial dilution of cell numbers. For example, for a six-well plate, we plated 1**×**10^4^, 5**×**10^4^, 1**×**10^5^ and 2 **×** 10^5^ A375 cells for overnight culture. Then cells were trypsinised and counted. The proliferation rate was estimated for 16 h. The confluency of cells for PLA assay should not be over ∼60%.

4. Leave the newly plated cells under the hood for 5 min with minimum vibrations to allow the cells to attach on the cover glasses. Incubate cells for 24 h at 37°C with 5% CO2.

**CRITICAL:** It is important to make sure that you have properly mixed the cells in each well, no obvious cell aggregates should be present in the cell suspension. The cells should be checked before the incubation at 37°C to make sure that the suspended cells are homogeneously distributed in each well. When this is confirmed, do not further shake the plate until the cells are attached. Non-homogeneous distribution of cells in the well leads to different cell densities at different positions, creating artificial differences in the number of eIF4F translation initiation complex in cells on the same well.

5. After 24 h (or overnight) growth, the cells can follow the subsequent steps, or can be treated with different perturbations (for example drug treatment).

**CRITICAL:** Before continuing the experiment, we suggest to check again under microscope to be sure that cell distribution is homogeneous --- no obvious cell clusters are present in each well.

### Fixation and permeabilization of cells

**Timing: 50 min**

**Figure 1:**
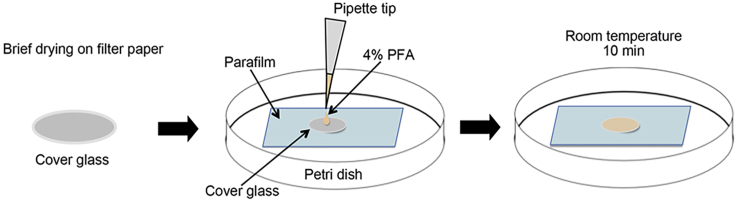
Fixation setup Spread parafilm on a Petri dish. Load the briefly dried coverslip onto the parafilm. Pipette 40 μL of 4% PFA solution on to the coverslip and stay at room temperature for 10 min. This allows minimum volume of solution used for the procedures and quick fixation upon removal of the samples from culture medium.

6. To remove the cover glass from the culture medium, we make a “L” shape of needle from an insulin syringe to detach the cover glass from the bottom, and take it gently with a surgical forceps avoiding to break the cover glass. Upon taking out the cover glass immediately after studied drug treatment, for example, combimetinib treatment in this protocol. Dry the cover glass on a piece of paper by touching the edge of the cover glass on a filter paper.
7. Prepare a slide covered with parafilms and place it in a 10 mm petri dish. Then place the cover glass on a pre-prepared parafilm. Add 30 μL 4% PFA immediately onto the cover glass under a chemical hood. Incubate for 10 min at room temperature (Figure 1).

**CRITICAL:** Translation initiation through the eIF4F complex is quickly regulated by different stress signaling cues. Therefore, quick fixation of the cells allows to minimizing the artifact induced by manipulation.

8. Prepare a 24-well plate by adding 1.5 mL 1**×** PBS in each well.
9. Recover the cover glass on a filter paper, remove the 4% PFA solution by touching the edge of the cover glass on a filter paper. Please make sure that the face of the slide with cells is exposed upwards. Draw a circle with an Immedge hydrophobic barrier pen around the cover glass. Place the cover glass in a prepared 24-well plate filled with 1.5 mL 1**×** PBS for 5 min at room temperature.
10. Gently remove the PBS solution with a vacuum pump.
11. To permeabilize the fixed cells, add 30 μL of 1**×** PBS Tween-20 0.2% solution for 10 min at room temperature.
12. Add 1.5 mL of a 1**×** PBS solution in each well without removing the 1**×** PBS Tween-20 0.2% solution and incubate for 5 min at room temperature.
13. Gently remove the 1**×** PBS solution with a vacuum pump.
14. Add 30 μL of the Blocking Solution (Sigma Aldrich, Olink, DUO92001) on each cover glass. Incubate for 20 min at 37°C.

**Pause point:** The cover glass with the blocking solution can be stored overnight at 4°C.

### Primary antibody hybridization

**Timing: 60 min**

15. To detect the eIF4E-eIF4G interaction, dilute the two antibodies eIF4E (anti-mouse) and eIF4G (anti-rabbit) 1:500 as the final dilution in the antibody diluent of the same tube. Alternatively, to detect the eIF4E-4EBP1 interaction, dilute the two antibodies eIF4E (anti-mouse) and 4EBP1 (anti-rabbit) 1:500 in the antibody diluent.

**CRITICAL:** The concentration and species source of the primary antibody are essential for the detection of protein-protein interactions, as we discussed previously. Therefore, to choose the appropriate antibodies for the PLA assay, one should perform in advance both a positive and a negative control. For example, we used the same dilution condition for eIF4E antibody alone or eIF4G antibody alone for primary antibody hybridization followed by proximity ligation probe hybridization with PLA probe Mouse Plus and PLA probe Mouse Minus for eIF4E detection; in the same sense, we used PLA probe Rabbit Plus and PLA probe Rabbit Minus for eIF4G detection. This will allow us to determine if the primary antibody is suitable for PLA assay. For a negative control in the aim of avoiding any non-specific background signals, we used two PLA probes Mouse Plus for eIF4E and two PLA probe Rabbit Plus for eIF4G. The same coupled oligonucleotides will not allow the ligation between them, therefore, any signals detected under this condition should be considered as non-specific background.

16. Add a solution of 1**×** PBS in each well without removing the blocking solution and gently/immediately aspirate with vacuum pump.
17. Add 30 μL of the corresponding primary antibody solution on the cover glass. Incubate for 1 h at 37°C.

***Note:*** Do not allow the sample to dry. Fill with a solution of 1**×** PBS in non-used wells within the 24-well plate.

18. Add 1.5 mL of Wash Buffer A in each well. Incubate on a shaker at low speed (For the shaker that we are using, the speed is at 64 rpm). Remove the wash buffer with vacuum pump and repeat this step twice.

***Note:*** The Wash buffer is conserved at 4°C. Before using, it should be recovered at room temperature during ∼30 min to reach the room temperature.

### Proximity ligation probe hybridization

**Timing: 60 min**

19. Dilute the two PLA probes Mouse Plus and Rabbit Minus stock solutions in antibody diluent to 1:5.

Alternate: the PLA probes could be applicable with Mouse Minus and Rabbit Plus.

20. Add 30 μL of the PLA probes mixed solution on the cover glass. Incubate for 1 h at 37°C.
21. Add 1.5 mL of the Wash Buffer A in each well. Incubate on a shaker at low speed (For the shaker that we are using, the speed is at 64 rpm). Remove the wash buffer with vacuum pump and repeat this step twice.

***Note:*** During the washing step, one should start to prepare the ligation solution at room temperature for the next step.

### Oligonucleotide ligation

**Timing: 45 min**

22. At the second time of the previous step, prepare the ligation reaction solution as follows:

**Table:** 

Component	Volume to add per reaction (μL)	Final concentration
5**×** Ligation buffer	390	1**×**
Ligase (1:40)	50	1 U/mL
Milli-Q water	1560	n/a

***Note:*** Ligase should stay on ice block. However, the prepared mix should stay at room temperature.

23. Gently remove the wash buffer A with a vacuum pump after the second time of previous step.
24. Add 30 μL of the ligation reaction mix on the cover glass. Incubate for 30 min at 37°C.
25. Add 1.5 mL of the Wash Buffer A in each well. Incubate on a shaker at low speed (For the shaker that we are using, the speed is at 64 rpm). Remove the wash buffer with a vacuum pump and repeat this step twice.

***Note:*** During the washing step, one should start to prepare the rolling cycle amplification solution at room temperature for the next step, and make sure that the amplification solution is protected from light.

### Rolling cycle amplification

**Timing: 70 min**

26. At the second time of the previous step, prepare the amplification reaction mix as follows.

**Table:** 

Component	Volume to add per reaction (μL)	Final concentration
5**×** Amplification buffer	395	1**×**
Polymerase (1:80)	25	1U/mL
Milli-Q water	1580	n/a

***Note:*** The amplification buffer contains fluorescent dyes and should be protected from light. The mix should stay at room temperature.

27. Gently remove the wash buffer A with a vacuum pump after the second time of previous step.
28. Add 30 μL of the amplification reaction mix on the cover glass. Incubate for 1.5 h at 37°C in the dark.
29. Add 1.5 mL of the Wash Buffer B in each well. Incubate on a shaker at low speed (For the shaker that we are using, the speed is at 64 rpm). Remove the wash buffer with a vacuum pump and repeat this step twice.

***Note:*** The plate should be protected from light during this step.

### Rolling cycle amplification washing

**Timing: 20 min**

30. Gently remove the wash buffer B with a vacuum pump.
31. Add 1 mL of 1:100 wash buffer B diluted in high purity water.

***Note:*** Users can process the samples at this step for further immunostaining by using routine immunofluorescence or probe-based methods. Please avoid to perform further immunostaining without washing at least once with 1:100 diluted wash buffer B since high concentration of buffer B may diminish the antibody staining efficacy.

32. Remove the cover glass on paper filter.
33. Air-dry during 15 min.

***Note:*** Protect from light. Alternatively, the cover glass can be further processed for cytoplasmic staining or co-staining with immunofluorescence. Please avoid overly drying which will decrease the PLA signal intensity.

### Slide mounting

**Timing: 15–30 min**

34. Add 2 μL of mounting medium with DAPI on each cover glass.
35. Reverse the cover glass to mount on a microscopic slide, gently press the top of cover glass with a syringe rubber to make sure that mounting medium spread all over the cover glass.

***Note:*** Avoid producing air bubbles in the process.

36. Seal with nail polish. Alternatively, if one uses ProLong Glass antifade Mountant (#P36980, Thermo Fisher), sealing with nail polish is not necessary.

**Pause point:** The slide can be stored at 4°C for long time before processing to microscopy image acquisition. In our laboratory, we have tested to store the slides for maximum 3 months at 4°C and we do not see obvious decrease of the fluorescence intensity.

37. Image acquisition after nail polish sealing for at least 30 min.

**CRITICAL:** Do not freeze the slide. Freezing at −20°C or −80°C will create large crystal particles in the mounting medium which will influence the image acquisition quality.

### Microscope setup and imaging

**Timing: variable 4–24h**

The following steps are described for automated slide scanning microscopic system. To be noted, manual microscope imaging is also suitable for eIF4F complex detection except that experimenters need to change each slide manually when the previous one is finished. We list the key parameters required for the image acquisition in the following tables:

**Table:** 

Parameter name	Parameter value	Note
Objective	20 X	Required for high throughput image acquisition
**Objective**	63 X oil immersion	Required for high quality 3D quantification
**Objective**	4 X	Required for the overview of the cell distribution and ROI selection.
**Z-stack**	3 z-stacks with 1 μm for each focus plane	Required for high throughput image acquisition with 20 X objective
**Z-stack**	20 z-stacks with 0.4 μm for each focus plane	Required for high quality 3D quantification with 63 X oil immersion objective
**Filter**	405 nm/450nm	Required for DAPI nuclear staining visualization
**Filter**	644 nm/670nm	Required for Cy5 far red PLA staining

38. Mount the slide onto the microscope stage:
  a. For large set of slide scanning and quantification of the relative number of translation initiation complex, we are using an Olympus Virtual Slide scanning system (VS120). This system allows fast acquisition of large data set with multiple levels of z stacks. Open the Control system VS-ASWFL on the computer. Select “Fluorescence Batch” button in the window of Batch Scan. The microscope will automatically scan the total number and positions of the slides.
  b. For quantification of the absolute number of translation initiation complex, we are using a True Confocal Scanner (TCS) SPE Leica confocal microscope, with multiple levels of z stacks covering the whole thickness of the cells in 3-dimensions.

***Note:*** The image acquisition process depends on the microscopy system. You may need to optimize your acquisition process by following the instruction of your proper microscope system.

39. Set up acquisition of z stacks, covering a thickness of the cells in an optimal way.
  a. In the case of quantification of the relative number of translation initiation complex, acquire 3 levels of z stack with 1 μm each. The Olympus Virtual Slide scanner will automatically focus on the cell plane by using DAPI channel. The other two z stacks will be −1 μm and +1 μm corresponding to the auto-focus plane.
  b. In the case of quantification of the absolute number of translation initiation complex, we acquire 20 z-stacks at each selected xy position in each wavelength channel with oil-immersion 63**×** objective, starting with Cy5, then DAPI. The distance between individual optical planes is 0.4 μm.

***Note:*** Illumination times should be adjusted for each slide to obtain optimal resolutions. Avoid excessive exposure time, since an observed saturation of pixel intensities will be detrimental for following image analysis. In particular, saturation of pixel intensities will artificially create point aggregates in the cells, this is not analyzable for the following quantification. In addition, overexposure will artificially decrease the number of spots counted by the software.

40. Acquire overview of the slide with 4**×** objective in DAPI channel with 150 ms exposure time in the Olympus Virtual Slide scanner system.

***Note:*** The following steps only apply to the quantification of the relative number of translation initiation complex by using Olympus Virtual Slide scanner.

41. Select at least 3 scan positions on each cover glass of the slide.
42. Process image acquisition with 20**×** objective and mirror cube QUAD, starting with DAPI channel (450 nm) with 3 ms exposure time, then Cy5 channel (644 nm/670 nm) with 300 ms exposure time at 3 z stack planes.

***Note:*** If you are using different fluorescent tag for the detection of proximity ligation assay, please optimize the corresponding filter. The PLA fluorescence are available for four different colors. Apart from the Cy5 far red color used in this protocol, three additional fluorophores are also applicable. For green fluorescence, one should use a 495nm/527nm filter; For Cy3 orange fluorescence, one should use a 554nm/576nm filter; and for red fluorescence, one should use a 594nm/624nm filter.

### Image process and computational analysis

**Timing: variable 2–24h**

43. Export image.
  a. Images acquired by Olympus Virtual Slide scanner are saved as VSI format. This format of image can be viewed with Olympus software OlyVIA. However, we adapted an ImageJ plugin module BIOP-tool (http://biop.epfl.ch/TOOL_VSI_Reader.html) for exporting TIFF images for following analysis. The detail manual of exporting images from VSI format can be found in BIOP-tool manual or on the website related to this protocol (https://sites.google.com/site/translationcomplexeif4f).
  b. Images acquired by TCS SPE Leica confocal microscope are saved as TIFF format. Readers can download Demo Images in DAPI channel and Cy5 channel with 20 z stack planes on the website (https://sites.google.com/site/translationcomplexeif4f). Open Images in the two channels as sequential images in ImageJ. Save the stacks of images as TIFF.
44. Image analysis.
  a. For quantification of the relative number of translation initiation complex, exported TIFF images can be opened with ImageJ software. Select Process/Binary/MakeBinary in the main menu of ImageJ to process the TIFF images into Binary format. After generating binary images, select Process/Filter/Median 2.0 in the main menu of ImageJ, followed by selecting Process/Binary/Watershed in the main menu to segment spots. You may process further by selecting Process/Binary/Find_Maxima in the main menu to exclude edge maxima, light background. The spot counting results are presented as medium ± standard deviation for 4 different images.
  b. For quantification of the absolute number of translation initiation complex, a MATLAB-based software, FISH-Quant (Mueller et al., 2013), is used for 3-dimension quantification of spots. Demo images and software setup can be downloaded on the website related to this protocol (https://sites.google.com/site/translationcomplexeif4f).

## Expected outcomes

**Figure 2:**
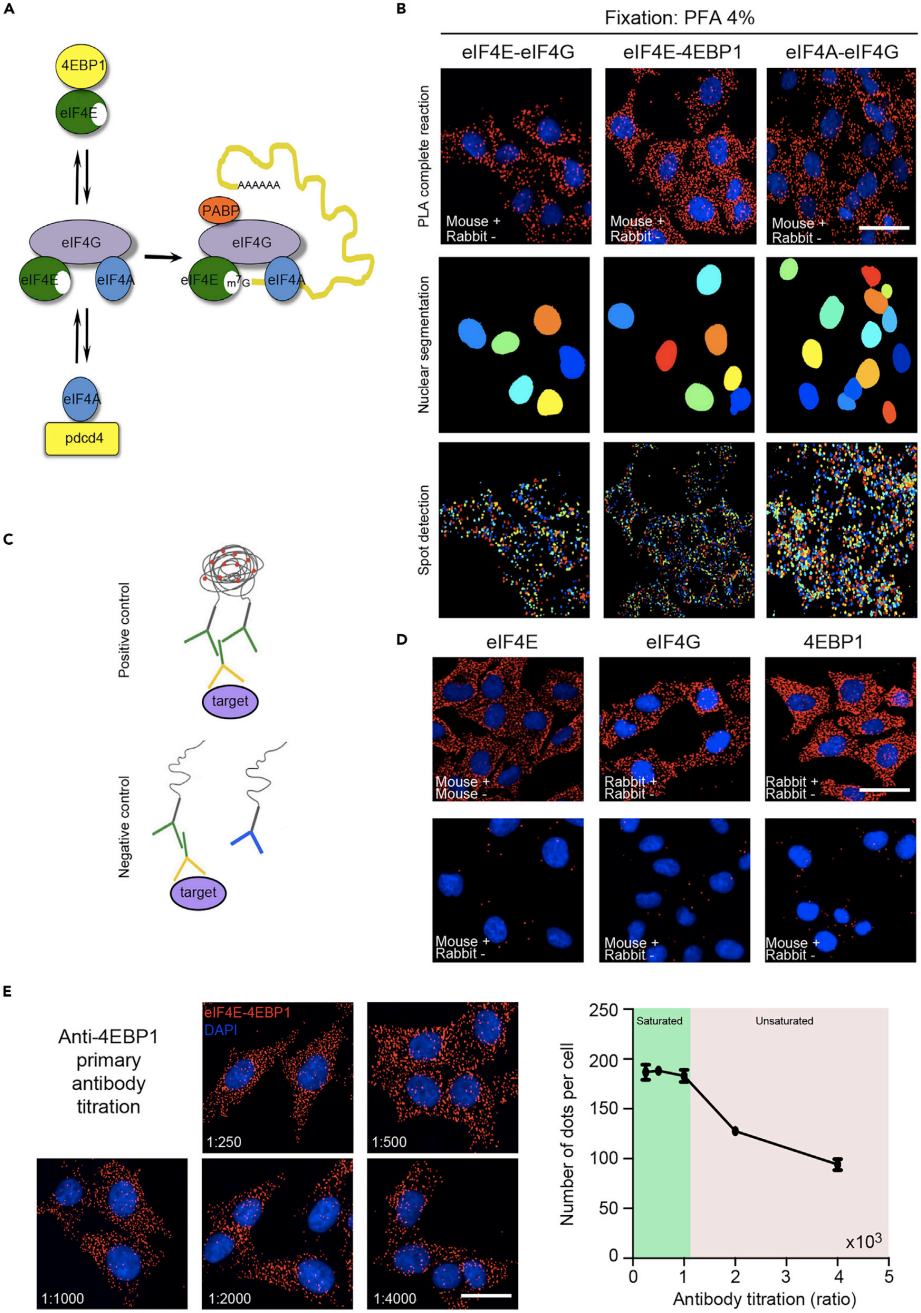
Optimization of the eIF4F complex detection by proximity ligation assay (A) Scheme of the translation initiation complex eIF4F. Inhibitory protein 4E-BP1 binds to eIF4E competitively with eIF4G to inhibit the formation of the eIF4F complex. Similarly, PDCD4 protein inhibits the RNA helicase eIF4A within the translation initiation complex. eIF4F recognizes the m7G cap structure at the 5′ untranslated region (5′UTR) of mRNAs, subsequently initiating the scanning of the 5′UTR. (B) Spots corresponding to single-molecule protein-protein interactions: eIF4E-eIF4G, eIF4E-4E-BP1 and eIF4A-eIF4G. Cells were fixed with 4% PFA. Nuclei was counterstained with DAPI. Middle panel shows the automatic detection of the nuclear mask, and the lower panel shows the automatic quantification of the relative number of spots. (C) The proximity ligation assay: the proximity of the DNA strands attached to secondary antibodies can be subsequently ligated by two other circle-forming DNA oligonucleotides. (D) Technical control should be performed in the aims of getting information about unspecific binding of primary antibodies. The anti-eIF4E antibody (anti-mouse), or anti-eIF4G (anti-rabbit), or anti-4EBP1 (anti-rabbit) is recognized by corresponding specie-specific PLA probes PLUS and MINUS. The background level is estimated by using two different specie-specific PLA probes (i.e., mouse PLUS and rabbit MINUS, bottom panel). (E) Titration of the primary antibody concentration (anti-4E-BP1). The number of spots shows antibody concentration-dependent effect; unsaturated antibody concentration underestimates the number of the translation initiation complex in the cells. In this case, the minimum dilution of the 4E-BP1 antibody is at 1:1000. Scale: 10 μm.

Translation initiation is regulated by two major events, including the formation of multifactorial 43S preinitiation complex (PIC) and the assembly of the eIF4F complex on the mRNA 5′UTR cap structure. The 43S PIC comprises 40S ribosomal subunit, multiple translation initiation factors (i.e., eIF1, eIF3, eIF5) and ternary complex consisting of initiator Met-tRNA (Sonenberg and Hinnebusch, 2009; Vogel and Marcotte, 2012). As a rate-limiting step of translation initiation, the assembly of eIF4F, a protein complex comprising the eIF4E cap binding protein, the eIF4G scaffolding protein and the eIF4A DEAD-box RNA helicase, recruits the 43S PIC via interactions with eIF3 (Chu et al., 2016; Jackson et al., 2010; Malka-Mahieu et al., 2017)(Figure 2A). The formation of the eIF4F complex is controlled by post-translational protein modifications (e.g., phosphorylation) and inhibitory protein-protein interactions (e.g., 4E-BP1 inhibits the formation of the eIF4F complex by competing with eIF4G to bind eIF4E.). Exploring and monitoring eIF4F formation has classically been based on indirect methods, largely dependent on bulk assays detecting phosphorylation of subunits of the complex, or on biochemical assays (e.g., cap-binding assay). We have recently described a procedure based on proximity ligation assay (PLA) to detect eIF4E-eIF4G and eIF4E-4EBP1 interactions at the single-molecule level in cell lines and in paraffin-embedded mouse and human tumors (Boussemart et al., 2014).

This protocol enables users to straightforwardly implement the detection of the eIF4F translation initiation complex in a variety of cells and tissues. It has various and wide applications. It can be applied to large-scale screening of the perturbation of interest on various cell lines, and can also be used to detect the translation initiation complex *in situ* in patient-derived biopsy tissue samples. In addition, this protocol enables one to quantify, for the first time, the absolute number of translation initiation complexes at the single molecule level in each observed cell(Ke et al., 2013), resulting in high-resolution studies of protein translational activity in cells and tissues*.*

The results of a typical *in situ* detection of eIF4E-eIF4G, eIF4E-4EBP1 and eIF4A-eIF4G interactions in the A375 human melanoma cell line are shown in Figure 2. Diffraction-limited spots are clearly observed by microscopy. Firstly, the relative number of translation initiation complex spots are recognized and counted by ImageJ software (Figure 2B). The specific signal (coming from the rolling circle amplification based on proximity of the PLA probes) must have a sufficient signal-to-noise ratio compared to the negative control (Figure 2C). In addition, the size of the signal spot should correspond to the size of a diffraction-limited spot (∼400 nm, in our case corresponds to ∼10 pixels). Finally, the signal spot should only appear in the corresponding fluorescence channel (i.e., in our case in the Far-red channel at 670 nm) (Figure 2D). It is noteworthy that inappropriate fixation or subsequent amplification may lead to an increase in the signals in cellular nuclei. This is often observed with 100% methanol or ethanol fixation at −20°C, or methanol/acetone fixation at room temperature (Boussemart et al., 2014). With saturated antibodies (see below), we detected an 80% increase in the amounts of dots in PFA-fixed cells compared to methanol/acetone-fixed cells (Figure 2B and Mendeley Data). We recommend experimenters to avoid alcoholic-related fixatives for the PLA detection. In this case, although signal spots could still be counted by our computational setup, the number of spots does not reflect the number of translation initiation complexes due to insufficient hybridization or amplification. In addition, the specificity of the complex detected by Proximity ligation assay should be tested further by siRNA targeting eIF4E, eIF4G or eIF4A (). For instance, the interaction between eIF4A and eIF4G is decreased following depletion of eIF4A or eIF4G1. However, depletion of eIF4E has no effect on the interaction between eIF4A and eIF4G. It is noteworthy that the quantification of the absolute number of the eIF4F complex is highly sensitive to primary antibody concentrations. A saturated concentration of primary antibody should be applied to the samples in order to be at a plateau period (Figure 2E). Further dilution of primary antibody would underestimate the absolute number of translation initiation complex in cells

**Figure 3:**
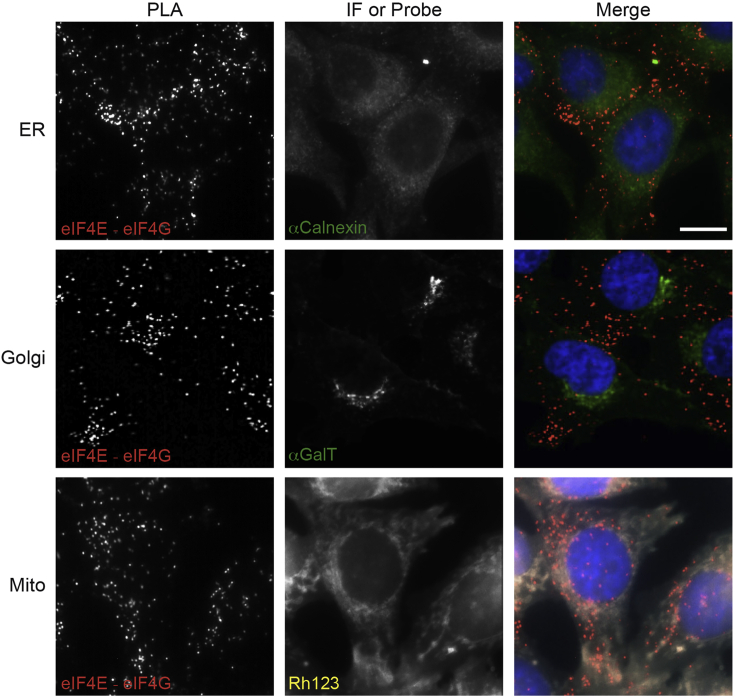
Subcellular localization of the translation initiation complex eIF4F Proximity ligation assay was performed for eIF4E-eIF4G interaction followed by counterstaining the corresponding cellular organelles. For endoplasmic reticulum (ER) and Golgi staining, anti-Calnexin and anti-GalT antibodies were used, immunofluorescent probes (IF) with fluorofore Alexa488 were used for the visualization. For detecting mitochondria (Mito), a chemical probe Rhodamine 123 (Rh123) was used for the recognition of the mitochondria in the cells. Nuclear was counterstained with DAPI.

Subcellular targeting of mRNAs has emerged as a major mechanism to establish functionally distinct compartments and structures (Besse and Ephrussi, 2008). Such mechanisms, capable of targeting protein synthesis in specific cell sub-compartments, are obviously important for proteins that can be deleterious when expressed ectopically. Being an *in situ* technology, this protocol not only allows the quantification of the absolute number of protein-protein interactions, but also the subcellular localization of the eIF4F complex. In our original procedure (Boussemart et al., 2014), we focused on the quantification of the number of interactions. In this protocol, we have further combined fluorescent probes or immunofluorescence co-staining of various cellular organelles with the proximity ligation assay. For instance, we used rhodamine 123, a cell-permeant dye that is readily sequestered by active mitochondria, to co-stain the mitochondria and the eIF4E-eIF4G interaction (Figure 3, bottom panel). Similar applications can be performed for other organelles, such as Endoplasmic Reticulum (Figure 3, top panel) and Golgi (Figure 3, middle panel) by using immunofluorescence with antibodies recognizing calnexin or GalT, respectively.

**Figure 4:**
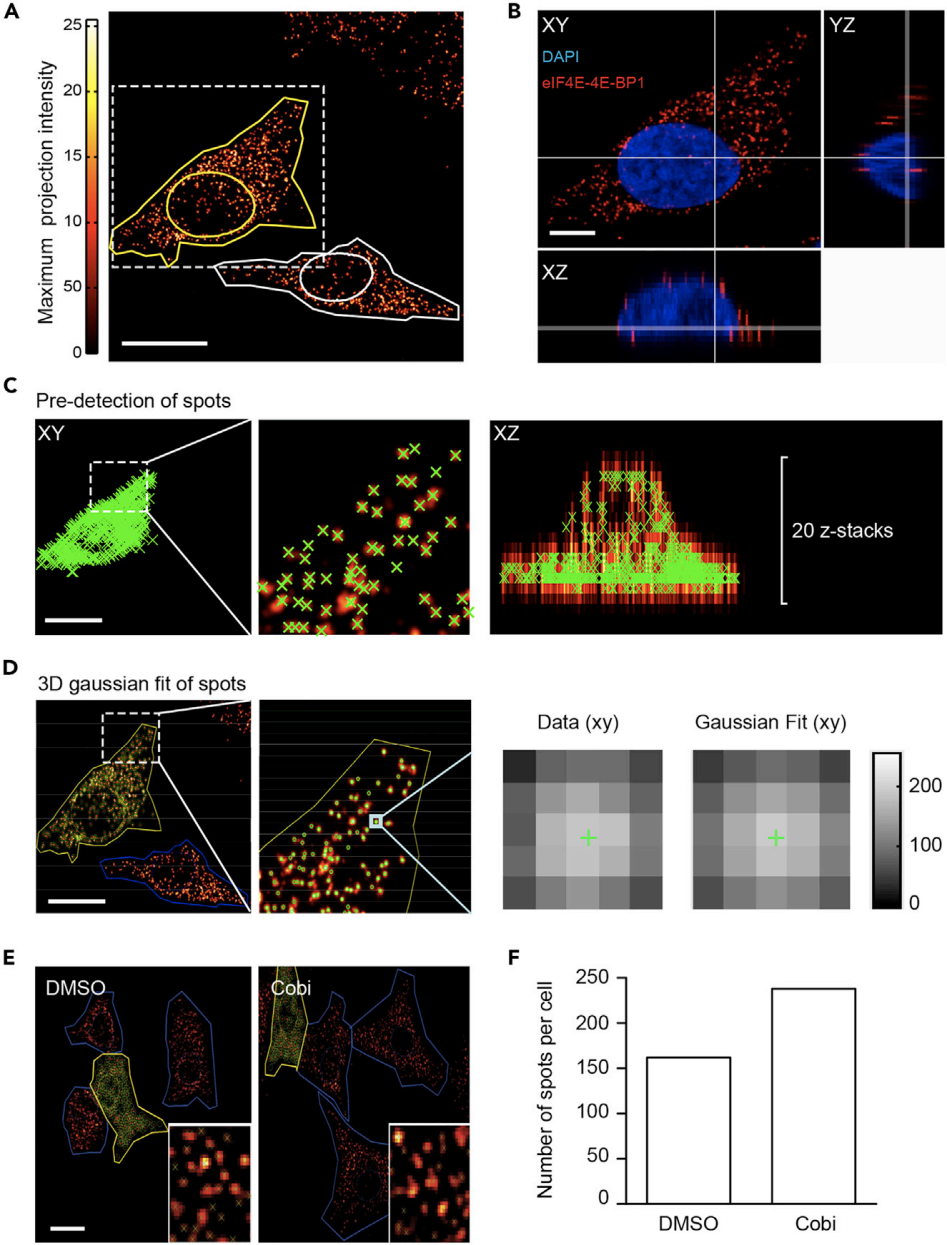
Quantification of the absolute number of interactions in three dimensions (A) A typical image of the PLA-based *in situ* visualization of the eIF4F complex with maximum intensity projection of the spots from confocal microscope scanned z-planes. Cell outline and nuclear mask were semi-automatically determined. Scale: 5 μm. (B) To determine the absolute number of the indicated interactions in individual cells, 20 z-planes were scanned with 0.4 μm distance between each plane and in total a 10 μm thickness of cells were acquired. Here three orthogonal axes were shown. Nuclei was counterstained with DAPI. Scale: 1 μm. (C) Automatic pre- quantification of the spots in 3-dimensions. XY and XZ frames were shown here. Green crosses indicate the detection of the spots by the program. Scale: 5 μm. (D) Pre-detected spots in 3- dimensions were then fitted to a gaussian model based on the intensity of each spot detected in each pixel, and the center of the spots were corrected by the gaussian fitting. This procedure largely separates those spots that localize in the same place or those that mistakenly quantified due to their diffraction between different z-planes. Scale: 5 μm. (E) A375 melanoma cells were treated with anti-MEK reagent (Cobimetinib, Cobi) for 24 h. DMSO was used as control. The eIF4E-4EBP1 spots were visualized with PLA-based assay, individual spots were quantified by previous procedures in 3-dimensions. Scale: 5 μm. (F) Upon MEK inhibition, an increase of the number of eIF4E-4EBP1 interactions between eIF4E-4EBP1 was observed in melanoma cells.

To quantify the absolute number of translation initiation complexes, the entire thickness of the cells of interest can be scanned by confocal microscopy (Figure 4A and 4B). For the A375 melanoma cell line, a distance of 0.4 μm between each z stack plane is sufficient for reconstructing 3D images and subsequent spot counting (Figure 4B). The 20 z- stack images are processed by ImageJ software, then 3D spots counting is adapted through a free MATLAB-based software, FISH-Quant (Mueller et al., 2013). Typical results of 3D spot counting are shown in Figure 4. The software firstly applies a two-step filtering process to reduce background noise and enhance the signal-to-noise ratio by using the Gaussian kernel method. The pre-detection of the PLA spots is thus performed on the filtered images on the corresponding *xy* plane of each z plane (Figure 4C). As shown in Figure 4D, pre-detection of spots will fit a 3D Gaussian algorithm to average images at sub-pixel resolution, which will shift the original image in such a way that the identified center of spot is truly the center of the image grid (Figure 4D right panel). Therefore, the algorithm can distinguish aggregated spots by calculating an image in the original resolution through averaging all values of the sub-pixels (see Manual of FISH-Quant software). However, the 3D quantification requires users to manually define the cell borders (cell segmentation), which is the most time-consuming step in the image analysis process. This computational method also allows to quantify the inactive eIF4E-4EBP1 complex at high-resolution level, which is challenging when using ImageJ software due to large number of eIF4E-4EBP1 complexes in cells. As shown in Figure 4E, we quantified the absolute number of eIF4E-4EBP1 complex in melanoma cell lines upon treatment with a MEK1 inhibitor (cobimetinib). Inhibition of MEK1 leads to a further increase the number of inhibitory complex eIF4E-4EBP1 in melanoma cells (Figure 4F), which is consistent with our previous report (Boussemart et al., 2014).

To be noted, the PLA-based detection of translation initiation complex at single molecule level can also be applied to patient-derived biopsy tissue samples, which may favor clinical diagnostics. We have developed a similar procedure for detecting translation initiation complex eIF4F in human melanoma tissue sections by using alternative staining markers. However, the patient-derived biopsy tissue samples are generally formalin fixed paraffin-embedded sections, thus additional antigen retrieval procedure is needed before undergoing PLA protocol. Following dewaxing and rehydrating the tissue sections, antigen retrieval is performed by heating the slides for 45 min at 95°C in citrate buffer, pH 6. Similar primary antibody concentrations are used for the detection of translation initiation complex eIF4F as described in the protocol, and the primary antibodies are incubated at 4°C overnight. After rolling circle amplification, the detection of the amplicons is carried out by using a 3, 3′, 5, 5’ – tetramethylbenzidine (TMB) chromogenic revelation, which appear as blue spot under a brightfield microscope and the cell nuclei are counterstained with Fast Red.

## Limitations

The first essential concern when implementing *in situ* single-molecule detection of the translation initiation complex is the integrity of the target complex in cells and tissue samples. The activity of eIF4F is highly sensitive to the status of cell intrinsic signaling and its extrinsic environment. The signaling pathways regulating protein synthesis interlink the protein synthesis machinery with cell surface receptors and cell-to-cell contact sensor proteins. It is therefore critical that the cells or tissues are dissected and fixed as quickly as possible and maintained at an intermediate confluence to avoid contact inhibition of cell proliferation. This requirement can become an issue when working on clinical samples, as this is a task that is often not performed or supervised by scientists familiar with the protocol. Another limitation is intrinsic to this protocol. As we are looking at translation initiation complex in fixed cells or tissue samples, this protocol can only reveal a snapshot of the status of eIF4F complex formation at a certain time point. Therefore, we cannot capture the temporal information on translation initiation complex formation as achieved when using the above-mentioned fluorescent reporter-based measurement of translation rate. Although multiple time points can be captured with this protocol to follow the changes in translation initiation complex formation in individual cells over time, the temporal dynamics of the activity of the translation initiation complex needs to be approached with mathematical models to evaluate the kinetic features of the underlying cellular protein synthesis mechanisms that are beyond the scope of the current protocol. However, the protocol described here will stimulate interdisciplinary scientists to develop such mathematical methods for the modeling and reconstruction of the dynamics of translation initiation complex formation. Although 3D image reconstruction using confocal microscopy scanning allows the quantification of the absolute number of the translation initiation complexes in individual cells as shown below, this quantification is limited to a certain number of cells depending on the size of the field of the scanning area and the computational capacity of the 3D spot counting, which is a limitation for statistical analysis. Large-scale scanning proposed by this protocol, like virtual slide scanning based on the Olympus system, facilitates the statistical analysis in a large population of cells. However, only the relative number of translation initiation complexes can be determined at this level.

## Troubleshooting

### Problem 1 (related to step: preparation of cells)

No cells or cells are not adherent to cover glass.

### Potential solution

Pre-treat cover glasses with 0.01% Poly-L-lysine(Boussemart et al., 2014).

### Problem 2 (related to step: primary antibody hybridization)

No PLA spots.

### Potential solution

Cell signaling antibodies can have a different concentration in different commercial batches. Adapt the dilution of your specific antibody with the concentration of the new batch(Ke et al., 2013).

### Problem 3 (related to step: fixation and permeabilization of cells)

High signal-to-noise background

### Potential solution

This is highly due to sample drying during the incubation. Add PBS 1**×** in empty wells in the same incubation plate, you may also need to redraw a circle with Immedge hydrophobic barrier pen during the experiment.

### Problem 4 (related to step: rolling cycle amplification washing)

High background or non-specific staining in nuclei.

### Potential solution

This may be due to wash buffer B which could be expired. Wash Buffer B can be stock at 4°C only during 1 month(Boussemart et al., 2014).

### Problem 5 (related to step: image process and computational analysis)

The FISH-Quant does not launch.

### Potential solution

This is probably due to the MATLAB version you are using. In this protocol, we uniformly used the MATLAB 7.12.0, R2011a and FISH-Quant version 1.0.

## Resource availability

### Lead contact

Further information and requests for resources and reagents should be directed to and will be fulfilled by the lead contact: Isabelle Girault (isabelle.girault@ose-immuno.com).

### Materials availability

This study did not generate new unique reagents.

### Data and code availability

This study did not generate any codes. The supplemental files are available at Mendeley Data: https://doi.org/10.17632/p4v35cdt9k.1
